# Bipolar Disorder: Identity, Social Support, Religiosity and Spirituality. Can Religiosity/Spirituality be a Mood Balancing Factor? An Italian Case Report

**DOI:** 10.1007/s10943-023-01991-5

**Published:** 2024-01-19

**Authors:** Jacopo Santambrogio, Jessica Maissen, Cosima Calini, Maria Cristina Filippo, Roberto Frigerio, Roberto Rampin, Giovanna Locorotondo, Adriana Pontiggia, Stefania Riboldi, Rossella Chiorazzo, Antonio Amatulli, Domenico De Berardis, Massimo Clerici, Simona Milani, Pietro Riccardo Cavalleri

**Affiliations:** 1 Adele Bonolis AS.FRA. Onlus Foundation, Vedano al Lambro, Italy; 2 Department of Medicine and Surgery, University of Milano-Bicocca, Monza, Italy; 3 Mental Health and Addiction Department, ASST Brianza, Vimercate, Italy; 4 Mental Health and Addiction Department, ASL Teramo, Teramo, Italy; 5 Mental Health and Addiction Department, IRCCS San Gerardo dei Tintori, Monza, Italy

**Keywords:** Bipolar disorder, Identity, Social support, Religiosity/Spirituality, Rehabilitation outcomes

## Abstract

This paper presents a case study to support the hypothesis that religiosity and spirituality (R/S), as mood balancing factors, could facilitate the recovery process for patients suffering from bipolar disorder (BD) once they have been stabilized and are receiving appropriate support (e.g., in a residential rehabilitative center). After a succinct review of BD and R/S, the patient’s medical history and rehabilitation pathway are described, with a particular focus on the role played by R/S. The authors found that in this case, once the patient was stabilized, R/S helped to consolidate her feelings of well-being, increasing her positive perception of social support services and ultimately her self-confidence.

## Introduction

The US National Institute of Mental Health has classified bipolar disorder (BD) as a severe mental illness (SMI) negatively impacting psychological/physical and social/occupational health; it is comprised of a complex group of severe and chronic mental health conditions that cause extreme mood swings. Both the Diagnostic and Statistical Manual of Mental Disorders (DSM–5) and the 11th version of the International Classification of Diseases (ICD-11) divide bipolar disorders into BD-I, BD-II and Cyclothymic, with diagnosis for BD-I being a past or present manic or mixed episode, for BD-II at least one hypomanic episode and one major depressive episode, and for Cyclothymic disorder, “at least 2 years (for children, a full year) of both hypomanic and depressive periods without ever fulfilling the criteria for an episode of mania, hypomania, or major depression” (APA, 2013).

BD usually requires life-long treatment, with lithium being generally considered to give optimum results as a mood-stabilizing agent (Buoli et al., 2020; Volkmann et al., 2020) although it has been suggested that there is a certain variability in the response (Papiol et al., 2022). As this illness often leads to functional impairment, long-term therapeutic interventions would appear to be essential to avoid psychotic symptoms, thus preventing relapses and preserving cognition (Bonnín et al., 2019). Although pharmacotherapeutic treatments are fundamental for effective recovery, an integrated approach including psychosocial interventions, individual psychoeducation, increasing physical exercise, compliance enhancement strategies and public health initiatives (McIntyre et al., 2020) is recommended.

Social support, defined by James House (1981) as “emotional concern, instrumental aid, information, and appraisal,” can influence clinical outcomes in people diagnosed with a bipolar disorder and appears to affect a broad range of biological and psychological processes. Many instruments have been developed over the years to measure social support: in particular, Cohen and Hoberman’s Interpersonal Support Evaluation List (ISEL) created in 1983 and validated in Italian in 2011 (Moretti et al., 2012) deserves a mention for its wide usage (Cohen et al., 1997; Cohen & Hoberman, 1983; Crane & Constantino, 2003; Hyman et al., 2003).

Although the effects of R/S on BD have not been extensively studied, available literature supports the beneficial effects of R/S and its coping mechanisms on both physical and mental health. During a 3-year follow-up among patients with chronic psychosis, Mohr et al. found that a positive use of religion can lead to a decrease in negative symptoms, an improvement in quality of life and better social functioning (Mohr & Huguelet, 2004; Mohr et al., 2010, 2011). The results of their literature review in 2011 led Barbara Pesut and her colleagues to suggest that R/S could be a relevant factor in managing BD and to conclude that it should be included in therapeutic pathways to develop well-being (Pesut et al., 2011).

While Jackson et al. (2022) found there is a lack of empirical research regarding R/S in BD patients, they noted from their scoping review that the measures of clinical outcomes indicate that intrinsic religiosity and religious beliefs and practices have highly positive correlations with improvement of bipolar disorder symptoms. They also reported that their participants felt that this area (even though sometimes difficult for them to discuss) should be taken into account by clinicians. The BD patient’s relationship with their self, with their identity, is a crucial issue in BD and R/S is an important factor in identity. There is clinical evidence to show that a full psychiatric anamnesis should include a patient’s spiritual history, as religion and spirituality are central components of the human experience and can influence the onset of mental illness and its course. Santambrogio et al. (2021) found there is increasing interest in the positive and negative impact of religion and spirituality on mental health, but like Jackson et al., the authors noted that little research has been done on how to approach these issues among people with serious mental illness (SMI). These authors also found that R/S can be simultaneously beneficial and detrimental for SMI sufferers; delusions and obsessions often contain religious content but this can also be seen as the search for a meaning throughout the course of a mental illness. These patients have the same religious and spiritual needs as the general population, and they seek the same support and meaning; many have histories of trauma and feel wounded in ways that religion and spirituality can help to address.

Religiosity is connected with mood symptoms and quality of life in BD. André Stroppa and his colleagues evaluated 168 BD patients with different scales in a cross-sectional study (Stroppa & Moreira-Almeida, 2013); the authors concluded that when patients have intrinsic religiosity and their religious beliefs and practices are positive, they tend to be less depressed and have a better quality of life, while the opposite is true when religious coping less strong. In a subsequent study with the same 168 individuals in a 2-year follow-up period, these authors concluded that, in addition to pharmacotherapy, R/S can provide a positive contribution to the state of mind of BD patients (Stroppa et al., 2018). However, another study with a sample of 189 Austrians and 180 Japanese, of whom a total of 120 diagnosed with BD, found spiritual well-being rather than religiosity was associated with resilience (Mizuno et al., 2017). Indeed, spirituality, in the sense of meaning, peace and purpose in life as opposed to religiosity or engagement with formal religious traditions per se, appears more closely linked to psychological resilience in patients with this disorder.

Mario Cruz and his colleagues (Cruz et al., 2010) noted that BD patients in mixed states tend to be more active religiously than patients in depressed, euthymic or manic states. In a review written in 2015, Pasquale De Fazio and his colleagues (De Fazio et al., 2015) noted that while R/S could be a psychosocial variable during the course of the illness, they found no support for the possibility of R/S providing protection or provocation in depressive or hypomanic phases; they also underline how different forms of R/S could reflect specific phases of BD, e.g., mystical and delusional thoughts may be present in manic phases that have psychotic elements (Pierre, 2001; Raab, 2007). From this point of view, R/S can provide an indication of the clinical state; however, mystic delusions must be distinguished from “spiritual emergencies,” defined by Alexander Moreira-Almeida and Harold Koenig as “critical phases linked with deep psychological changes with unusual experiences involving conscience, affectivity, thought, sense-perception and physical symptoms” (Moreira-Almeida & Koenig, 2006).

The majority of the articles examined in the course of writing this article express the need for further study of the relationship between BD and R/S.

## The Case Study

### Psychiatric History

This study examines the case of P., an Italian woman born in 1962 with a long history of mental disorder. At the time of writing, she was undergoing treatment at the “Adele Bonolis—Fraternal Assistance Foundation” (AS.FRA.), a residential care center for patients with mental disorders. The Foundation considers religion to be important, and its mission specifies that its work is inspired by the principles of Christian charity. This study focusses on the development of P.’s illness from when she entered AS.FRA.’s intensive care unit in 2018 till 2021.

P.’s father died when she was 18 years old and it is probable that this event was the catalyst for her depression. She was first hospitalized in 1999 at the age of 37 following several panic attacks and was diagnosed as having an “unspecified neurotic disorder.” She was in psychoanalysis from 2002 to 2005, but her symptoms became more evidently psychotic. In 2006, she was finally diagnosed as BD–I with mood-congruent psychotic features and several medical comorbidities: hypothyroidism secondary to psychotropic drugs in replacement therapy, schwannoma, macular dystrophy, mammary dysplasia, uterine myomas, constipation. Other hospitalizations followed and in 2015 she was admitted to three different rehabilitative facilities and was described as anhedonic, asthenic, apathic, with obsessive, delusional thoughts, and auditory hallucinations. She attempted suicide and was compulsorily hospitalized. In 2017, she suffered a period of dysphoria, impulsiveness, and disinhibition, after which she stabilized into depression. In 2018, she entered a high assistance rehabilitation program in AS.FRA.

P. is well educated, having obtained a degree in architecture, but she has never been employed and has a full disability pension. She was married for 4 years but she separated from her husband in 2000 and is now divorced. She has two older sisters.

### Rehabilitation Program

During her first clinical assessment at the beginning of March 2018, P. defined herself as a bipolar patient, indicating that she has identified with the disorder. She was oriented, collaborative, had taken care with her appearance and her mood was balanced; she was talkative, coherent, and understandable, and her thoughts were correct in form and content.

A support program was designed, encouraging her to consider an autonomous future in her own home; however, she was worried about her ability to function mentally, about her finances and living alone. During this period, she participated in many of the activities offered by AS.FRA such as theater, ceramics, sewing, but was unable to overcome the financial concerns and fears of being left alone. She showed good introspective abilities (probably due to her previous experience with psychoanalysis) and transference. She asked for the dosage of the psychotropic drugs she was taking to be reduced, because “she felt fine.” During the first period of the treatment, the psychiatrist acquiesced, gradually reducing and finally eliminating the dosage of Amisulpride. However, she was not able to overcome her fears and asked to remain in the Community as long as possible.

During the second semester, her condition deteriorated: at the end of September 2018, she showed symptoms of reference exacerbated by anxiety; this occurred particularly after she had spent time in her home. She felt confused, as evidenced by the statement, “I hear voices: ‘why are you cleaning your teeth?’ but it could be a mother saying it to her child in the apartment next door,” but she was also proactive, planning to renew her driving license and get a car. In this period, she attended the AS.FRA. Foundation theater group, taking a leading role in “Hamlet,” which indicated an increase in her self-confidence, but by the end of October 2018, she was dysphoric and reported disrupted sleep, claiming to feel rested after only three hours sleep. When her relatives visited, she was more talkative than usual and demonstrated persecutory behavior; her thoughts were tangential and disorganized. At the end of this semester, her YMRS score had increased to 37, indicative of moderate mania. The following utterances are indicative of her state at the time: “I am a Zen monk” (this is in line with Ouwehand et al.’s (2017) comment that persons with BD may have religious psychotic symptoms in the manic phase, “What you call a manic phase is a necessary state of being for me,” “I hear my relatives’ voices, but maybe they are only in my thoughts.” Her medication was adjusted, with Quetiapine being titrated up to 800 mg/day, Lithium Carbonate up to 1200 mg/day, and Amisulpride was reintroduced up to 200 mg/day.

In January 2019, she recovered from the manic phase and her mood became more stable with some depressive features. Her treatment was adjusted, downgrading Quetiapine to 600 mg/day and Amisulpride to 150 mg/day. She spent Easter in “Vangelo e Zen” (“Gospel and Zen”) an inter-religious community founded by a Xaverian missionary and a Zen Soto monk who have combined Christian principles, rituals, and tradition with those of Buddhism in a constant dialog. She participated actively in this community and became close to its leaders, because she felt “embraced by a father” and drew spiritual nourishment and peace from the meditation. Commenting on the possibility of going back home, she said: “I don’t want to, because there are Christians and not Buddhists in the household,” which indicates that, in line also with Jackson et al.’s findings, she was having difficulty in integrating her R/S experiences in a condition of psychic stability. Her treatment was adjusted again, substituting Lithium Carbonate with Lithium Sulfate, 83 mg, 2 + ½ tabs, for a more balanced distribution. She was given regular ECGs and her Lithium dosages were normal. She seemed to lose interest in the idea of renting the house, mainly because she was fond of it and had good memories; finally, in May 2019, for the first time, she said she was contemplating the idea of going back home. On her evaluation at the end of the first semester of 2019, she scored 0 on YMRS, indicative of remission, and her score on MADRS had increased to 21, indicative of moderate depression. However, she participated in various cultural projects such as a vacation in Trieste, which reassured the clinician that the risk she would attempt suicide was manageable. In this period, she recalled her previous relationships and her uninhibited rapport with her own body; this caused her to feel a sense of guilt so she turned to religion for forgiveness, maintaining that spirituality had always been present in her life and had offered strong support during her illness. When hospitalized, she went to the chapel to say the rosary and participate in the functions, while at AS.FRA. she helped the chaplain prepare for Mass and she did the readings. She remembered feeling disturbed by the Eucharist transubstantiation during her manic and psychotic phases. At that point in 2019 however, her Christian spirituality was characterized by a mood of quiet depression; she saw sin as something overwhelming, like a mystic. Nevertheless, she reported being very close to God, to Jesus Christ and his mother Mary, and considered her rehab program to be “the atonement for my sins.” On the 2019s semester test evaluation, the YMRS score was again indicative of remission and at 14 the MADRS was indicative of mild depression.

During her first psychiatric visit in 2020, she was melancholic, saying that “winter makes me depressed.” She imagined a future of sadness in her house, foreseeing deprivations, “I will live in the cold.” However, she also expressed a different perspective, imagining a future of hope, linking it to a spiritual dimension, “If I will be poor in spirit, I will join Christ.” For the first time during a psychiatric session, she explained clearly how the spiritual dimension and the Catholic faith helped her look to the future with hope. Her psychiatric history and future projects found meaning in a common ground of sacrifice and resurrection, the experience of Christ. At the end of the first semester 2020, her YMRS value was 0 once again, and MADRS was 12, indicative of mild depression. During the psychiatric visits in August 2020 and later, her moods were balanced and she was receptive to the future. She spoke about her religious sensitivity, as a search for meaning in her life, a search for a plan given by God, even if she felt “on the melancholic side,” perceiving “a hole in reality” and on the second semester 2020 test evaluation, her YMRS score continued to be 0, and the MADRS rating had decreased to 7, indicative of very mild depression.

At the beginning of 2021, a specific psychological pathway was added to the weekly psychiatric sessions to support her decision to go home, and to work on the steps needed to achieve this goal. At this point, P. focused her thoughts on her home and the relationships she had had during her manic phases, comparing them to the actual situation in which she felt “lifeless,” but at the same time was engaged in relationships “in a platonic way.” She was happy to continue to be a member of the group of patients involved in the organization of the weekly Mass with the chaplain. She felt sustained by the priest because he assigned her responsibilities and, like the priests in the Gospel and Zen community, he represented “a father figure.” She said she felt anxious and thoughtful before the Mass, then “during the Mass something happens, the Eucharist, and I feel better.”

On June 22, 2021, it was decided that P. should start spending short periods (3-night stays) at home during the summer. After these “breaks from the institution,” she reported feeling good, because she was looking after her house and she had her own time and space. Some friends were helping her with the housework; she said she felt “fulfilled.”

To summarize, on the first semester evaluation in 2018, when she was in a stable clinical phase, she scored 46 on the ISEL set, but on the next assessment, when she was in a manic phase, she scored 61, indicating that the perceived social support could have been higher, but the data were probably affected by the instability of her moods. At the end of 2019, when she was depressed after the manic phase but had had pleasant experiences, she scored 49 on the ISEL. By 2020, her clinical condition had stabilized and at the end of the year her ISEL evaluation was 63, indicating that her perception of the support she was receiving was much improved. On the last evaluation in June 2021, her overall ISEL score was 77, as her scores had considerably increased compared with her ratings on the first semester 2018 (see Table 1 below).

**Table 1:** Test results—1 semester 2018 to 1 semester 2021

Test	Score S1-18	Score S2-18	Score S1-19	Score S2-19	Score S2-20	Score S1-21
YMRS	18	37	0	0	0	7
MADRS	8	6	21	14	7	7
ISEL (Moretti et al., 2011)	46	61		49	63	77
Appraisal Support subscale	16	23		12	18	23
Tangible Support su bscale	11	12		13	15	17
Self Esteem Subsacle	7	10		10	14	19
Belonging Support Subscale	12	16		14	16	18
Durel (Koenig & Bussing, 2010	19	17		21	25	22
ORA (Koenig & Bussing, 2010)	5	5		6	6	5
NORA	2	2		2	6	5
IR	12					12
WHO-QOL	48.4					56.4
1. Physical Health	12					13.2
2. Psychological Health	12.8					14.8
3. Social Relationships	12					14.8
4. Environment	11.6					13.6

*YMRS*: Young Mania rating scale; *MADRS*: Montgomery and asberg depression rating Scale; *ISEL*: Interpersonal support evaluation; *DUREL*: Duke University Religion Index; *ORA*: Organizational religious activities; *NORA*: Non-organizational religious activities; *IR*: Intrinsic religiosity; *WHO*-*QOL*: World Health Organization-Quality of Life

## Discussion

During her years in AS.FRA., P. participated in the “Gospel and Zen” community and attended weekly Mass, as religion was important to her. During her manic phases in 2018, she had spiritual experiences that could be defined as visual hallucinations; moreover, she remembers standing in her garden, dressed in a kimono, listening to voices, the voices of her neighbors but also the voices of plants and God. In that period, her scores regarding participation in organizational and non-organizational religious activities remained unvaried, but the measurement of intrinsic religiosity decreased (from 12 to 10). In fact, mood instability caused her to feel insecure about the presence of the Divine, which underlies her whole approach to life. She also had delusional thoughts related to her participation in the “Gospel and Zen” community (“I am a Zen monk”). When she was depressed, she perceived religion as a way to atone for her sins. It is interesting to note that when P. took her medication as prescribed and attended her psychiatric visits regularly, her approach to R/S became less obsessive and healthier, and a more integrated aspect of her identity (Johnson et al., 1999). In 2019, when her clinical condition improved, her participation in organized religious activities increased (from 5 to 6) and so did intrinsic religiosity (from 10 to 13). During 2020, the year most affected by the COVID-19 pandemic when outside activities were forbidden, she spent more time in the community and her participation in non-organizational religious activities increased, e.g., reciting the rosary, Bible reading and meditation (from 2 to 6). She was able to participate in the “Gospel and Zen” community activities and attend weekly Mass at ASFRA with a clearer consciousness. Her R/S beliefs consolidated and matured in the ambit of the inter-religious community led by the two priests and the religious activities connected to the preparation of the Mass with the officiating priest in AS.FRA. Indeed, an interplay between the R/S dimensions and the perception of social support could be assumed. Having a role in this community and participating in these activities reinforced her faith; it became more tangible to her and therefore she perceived it as more supportive (Montgomery & Åsberg, 1979). The improvement in R/S scores could be related to the improvement in overall ISEL score, in particular in the Appraisal Support Subscale. Moreover, her association with the priests in their role as authoritative religious figures, helped her to feel embraced by a father figure. Since the onset of P.’s psychiatric disorder can be collocated as following the death of her biological father, experiencing a bond with a father figure (be it the Deity or human fathers, the priests) in the context of R/S provided an opportunity for healing. In 2021 all her R/S scores decreased slightly, as she developed a more balanced approach to this dimension.

With regards to clinical stability, P.’s mood became more stable over the 3-year follow-up. She arrived in a hypomanic state in 2018, became depressed in 2019 and then balanced from 2020 until the end of this report. She developed a deeper awareness about her condition and the need to follow medical prescriptions, “I feel good because I take these medications,” she reported, as opposed to, “I feel good, so I can reduce or stop medications.” This led P. to modify her requests, as she now then understood that proper therapy helped her have a more balanced relationship with reality, and she no longer experienced mood instability, emotional outbursts, and psychotic symptoms. She was able to participate in many activities and have healthy relationships. Moreover, since 2018, she perceived the activities and the group to which she belonged were a way “of stopping the mind from thinking about her financial situation.”

Over the years, P. has started to feel recognized and valued in a wide range of group contexts, because she was able to express her cultural and social abilities. She also experienced satisfaction in sharing time and experiences with friends and significant others. As her psychiatric and psychological pathway progressed, P. became increasingly confident about the possibility of going back home to live and her perspective became more autonomous. She saw that friends and relatives supported her decision and were available to help her, even in practical situations regarding the house.

## Limitations

In general, the limitation of single case studies is the difficulty of applying the findings to a wider population. However, we considered that the results from the evaluations from this case and the diagnostics could provide a useful contribution to a field where relatively little research has been done.

## Conclusions

P.’s psychiatric history is complex and although she has accepted the diagnosis of BD, even identifying with it, finding the correct balance of psychotropic medication and social care has been challenging. Her fears for the future and lack of self-confidence led her to request comprehensive care and hospitalization, despite being financially autonomous and with a home of her own. In the long term however, the psycho-dynamically oriented psychiatric pathway of her rehabilitation program is helping her to become more self-confident and receptive to the idea of having a future in her own home. Her case shows how the clinical features of BD are connected not only to medical aspects but also to the psychosocial dimensions such as rehabilitation treatment (Colom et al., 2003), social support (Johnson et al., 1999) and R/S that constitute her identity.

In this case study, we have seen that when the patient’s mood was stabilized, R/S was an important factor that contributed to maintaining and increasing this stability, leading the patient to be more receptive to experiencing R/S positively in physical contexts (e.g., religious groups), which increased a positive perception of social support, further consolidating stability.
